# The flow of corporate control in the global ownership network

**DOI:** 10.1371/journal.pone.0290229

**Published:** 2023-08-24

**Authors:** Takayuki Mizuno, Shohei Doi, Shuhei Kurizaki

**Affiliations:** 1 Information and Society Research Division, National Institute of Informatics, Tokyo, Japan; 2 Public Policy School, Hokkaido University, Sapporo, Hokkaido, Japan; 3 School of Political Science and Economics, Waseda University, Tokyo, Japan; Lodz University of Technology: Politechnika Lodzka, POLAND

## Abstract

We propose a model and algorithm to measure the amount of influence a shareholder has over the flow of corporate control held by the ultimate owners. Existing models of corporate ownership and control either focus on the ultimate owners’ influence or inadequately evaluate the influence possessed by intermediate shareholders in a ownership network. As it extends Network Power Index (NPI) that describes the the power of corporate control possessed by the ultimate owners, our new model, Network Power Flow (NPF), delineates the distribution of ownership influence among shareholders across the network and identifies the channels through which the ultimate owners’ corporate control travel through the global shareholding network. Our analysis of NPI and NPF values for 7 million ultimate owners and 16 million shareholders reveals a new landscape of ownership and control in the global shareholding network that remained opaque before.

## Introduction

The rapid growth of the equity market in recent years is shifting the distribution of corporate control in the global financial network. A driving force behind this growth is the rise of passive index funds. In one estimate, the assets under management in the global equity market have reached 100 trillion USD in 2021, equal to 250% of the combined GDPs of the U.S. and China. It is also estimated that the global equity market will increase by nearly 50 percent in size by 2025, reaching $145 trillion [1, 2]. While any entity gaining influence in this “machine” will be bestowed with the enormous power to control the global economy and beyond, Fichtner et al. report that the American “Big Three” asset managers, BlackRock, Vanguard, and State Street, dominate the influx of capital into the market [3].

This observation suggests that these asset managers may play a powerful role in the global ownership network. Yet, the existing studies are not equipped with adequate measures or models to describe their ownership influence [4–7]. In particular, most studies measure the ultimate owners’ power to control companies, but the asset managers are not the ultimate owners; they are *intermediate* shareholders that are also owned by their ultimate owners.

Moreover, some of the large asset managers are widely held companies whose ownership is often dispersed among many, less influential shareholders. And yet, they have substantial influence. How do they collect the power of corporate control? An adequate measure should account for a peculiar mechanism in the ownership network that amplifies their ownership influence through the indirect controls of fragmented voting rights attached to dispersed ownership.

This article fills this gap by proposing a new model and algorithm to measure the influence possessed by intermediate shareholders in an ownership network. To understand the source of intermediaries’ influence, we develop a model that allows us to assess how the influence possessed by intermediaries relates to that of their ultimate owners. In doing so, we extend our previous study on the measurement of the ultimate owners’ power of corporate control to measure intermediate shareholders’ power [7].

Our previous article introduces the model, called *Network Power Index* (NPI), to measure the power of corporate control possessed by ultimate owners in the shareholding network. NPI follows the literature on collective decision-making to define the power as the probability that an ultimate owner becomes pivotal in forming a majority to dominate decision-making in a target company. NPI extends the Shapley-Shubik (SS) power index, a canonical model of voting power in a *weighted majority voting game*, in the context of the ownership network [8, 9]. Since the SS power index concerns a single voting game of majority formation, it only measures a voter’s “direct” control over the majority in a given company. On the other hand, NPI accounts for coalition-building through a network of multiple weighted voting games that are interdependent and sometimes nested in other games. This forces us to take into account the transitivity and consolidation of fragmented voting power both upstream and downstream in the ownership network if we want to assess the formation of either direct or indirect control of companies.

Building on NPI, this article introduces a model, called *Network Power Follow* (NPF), to measure the amount of corporate control possessed by ultimate owners flows through an intermediate shareholder in the ownership network. A shareholder’s NPF is defined as the probability that the shareholder becomes pivotal in the path(s) of links that establishes a majority share in a target company. Since NPF is an extension of NPI, it captures the indirect control that may be dispersed among fragmented ownership. While NPI is interpreted as the size of the power to control companies, NPF is considered to quantify how much influence a given shareholder has over the “flow” of corporate control in the network. In this way, we have an intuitive method to compare corporate influence possessed by ultimate owners to that of other shareholders.

We first review our original NPI for the ultimate owners and then introduce the NPF model for the intermediate shareholders. In doing so, we draw some comparisons to other existing measures of the flow of influence in networks. We then describe our algorithm that implements our model in real-world data on the global ownership network. The complex network of corporate ownership generates some interesting features. Among other results, we show that some state governments outperform institutional investors and asset managers in obtaining influence in the global economy. Since the influence of “statist” governments is not confined to the control of their state-owned enterprises, our results corroborate the rise of state-owned investors (SOIs) in the global ownership network on one hand [10], and suggest their transnational “non-business” objectives on the other hand [11].

## A model of corporate control

**Fig 1:**
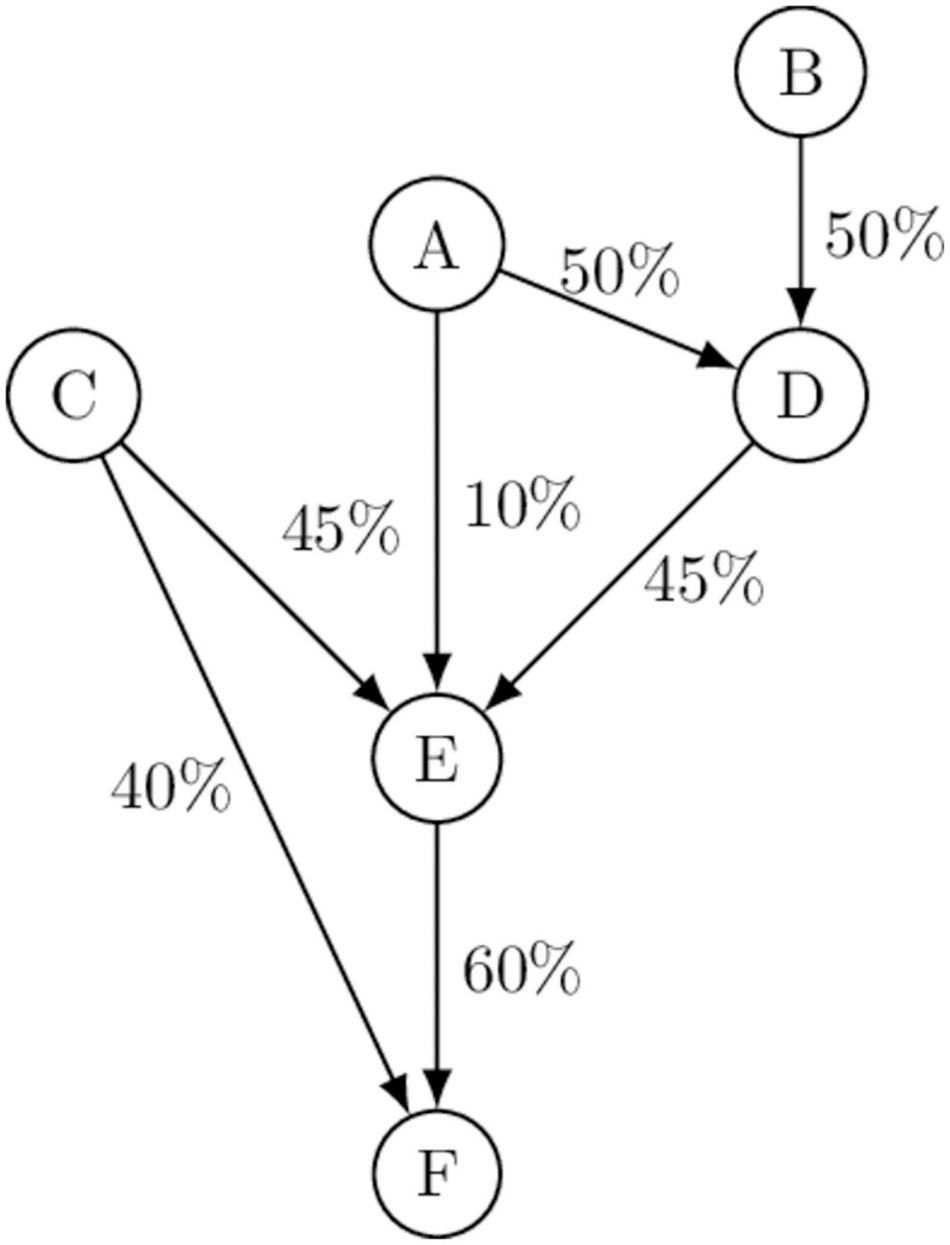
Stylized ownership network.

We first use a stylized example to develop an intuition for the power of corporate control possessed by intermediate shareholders. Consider the ownership network depicted in Fig 1, which consists of three ultimate owners, *A*, *B*, and *C*, who may own shares in three companies, *E*, *D*, and *F*. Ownership relations are represented by directed edges, and the share of voting rights are indicated along each edge.

Ultimate owner *A*, for example, holds indirect ownership in company *F* through its direct ownership in *D* and *E*. In terms of *A*’s indirect ownership in *F*, companies *D* and *E* are intermediate shareholders who may act on behalf of *A* so that *A* can exercise ultimate control over *F*. As such, the intermediate shareholders are crucial to the power that *A* may have in the network in at least two ways. First, intermediaries *D* and *E* are indispensable for *A* to construct an ownership structure that enables *A*’s indirect control over company *F*; without *D* or *E*, *A* would not be able to establish the control path(s) towards *F*. Second, intermediaries *D* and *E* may also gain enhanced power vis-a-vis *F*, more than their respective power alone, by aggregating their ultimate owner’s power (where an intermediary is assumed to act on behalf of its ultimate owner) [12].

Note that a shareholder’s possession of the power to control a target company does not necessarily imply the shareholder always dictates target company’s corporate decision-making. As we describe below, our definition of power follows a commonly adopted definition in social sciences; that is, *A*’s power over *B* is *A*’s capacity to influence *B*’s behavior [13].

In what follows in this section, we characterize the power of corporate control in an ownership network for intermediate shareholders as an extension of the power possessed by the ultimate owners.

### Ownership shares and control

To define the power of intermediaries, we first describe the power of the ultimate owners in the network who may delegate their power to, or collect power from, the companies in which they have ownership (such as their subsidiaries). Recall that in our stylized example in Fig 1 shareholders *A*, *C*, and *D* respectively possess 10%, 45%, and 45% shares in company *E*. If all shares equally come with the voting rights, voting power is allocated among these shareholders in proportion to the size of direct ownership in *E*, that is 0.1, 0.45, and 0.45 for *A*, *C*, and *D* respectively. However, the proportion of the voting rights (or the proportion of the ownership shares) does not linearly translate into corporate control, since a company’s managerial decisions typically require a majority or other threshold (i.e., a quota).

The manner in which each shareholder’s ownership is converted into power over corporate decision-making is best described as a weighted voting game among the shareholders with a quota. In this game, a shareholder or a group of shareholders who collect more voting rights than the quota prevails in a company’s decision-making. The Shapley-Shubik power index is the best workhorse model of a generalized weighted voting game, where a player’s voting power is defined as the probability that the player can be pivotal across winning coalitions in a voting game [8, 9]. A permutation of players forms a winning coalition if their voting rights collectively meet the quota but fails to do so if any of its members withdraw itself from the coalition. In Fig 1, each of six permutations (or coalitions), {*A*, *B*}, {*B*, *A*}, {*B*, *D*}, {*D*, *B*}, {*D*, *A*}, and {*A*, *D*} forms a winning coalition in decision-making over *E*, and the last shareholder in each coalition is pivotal. Each of *A*, *B*, and *D* appears twice as a second member among the six permutations, suggesting that they all have the same probability, 1/3, of being pivotal in controlling *E*’s decision making, even though their respective voting weights are different.

### Network power index

We now embed the Shapley-Shubik power index in the network of corporate ownership where a multitude of corporate decision-making bodies (i.e., weighted voting games) are stratified and interconnected. This allows us to examine how a change in ownership structure upstream in the shareholding network may change the distribution of voting power among the shareholders. To see this, suppose that shareholders *A* and *B* each own 50% of the voting right in company *D* as indicated in Fig 1. If shareholder *A* happens to prevail in *D*, it forms the majority in *E* by consolidating its own voting rights in *E* and *D*’s voting rights in *E* (i.e., 10% + 45% = 55%). Alternatively, if shareholder *B* happens to control *D*, it may also indirectly exert its influence over *E* through *D*. As such, in an ownership network, shareholders may possess more capacity to control their target companies than their direct ownership shares alone would suggest as the network structure allows for consolidating indirect controls to form a winning coalition in a target company. We have proposed a model, called the Network Power Index (NPI), to describe exactly this idea for ultimate owners in the global ownership network [7].

Similar to the Shapley-Shubik power index, NPI is defined as the probability that an ultimate owner can be pivotal in forming a majority coalition in the decision-making of a target company, where a majority coalition can be formed indirectly through intermediate shareholders. For example, if ultimate owner *A* dominates in *D* with probability 1/2, *A* obtains full control over *E* by commanding *D*’s voting rights in *E*. In contrast, if ultimate owner *B* controls *D*, another ultimate owner *A* has probability 1/3 of controlling *E*. In sum, the overall probability that *A* controls *E* is given by $\frac{1}{2}\times 1+\frac{1}{2}\times \frac{1}{3}=\frac{2}{3}$. In a similar fashion, the NPI for *B* and *C* over *E* are $\frac{1}{6}$.

### Network power flow

The NPI describes the distribution of power of corporate control in the ownership network, but it is not designed to assess the power that intermediary shareholders may possess despite the fact that intermediary shareholders are essential for their ultimate owners to form their ownership control. For example, while ultimate owner *A* is most influential in our running example in Fig 1, its control can be extended to *E* and then to *F* only because *D* possesses 45% voting rights in *E* which contributes to *A*’s indirect control over *E*. Furthermore, if any ultimate owner was to indirectly control *F*, that control would have to be channeled through *E* and utilize *E*’s 60% share in *F*. The NPI does not describe the role that intermediate shareholders may play in establishing the ownership path(s) linking their ultimate owners to the target companies downstream. This motivates us to propose a model, which we call the Network Power Flow (NPF), that describes the power that intermediate shareholders may have in shaping the distribution of corporate control in the ownership network.

#### Examples

**Fig 2:**
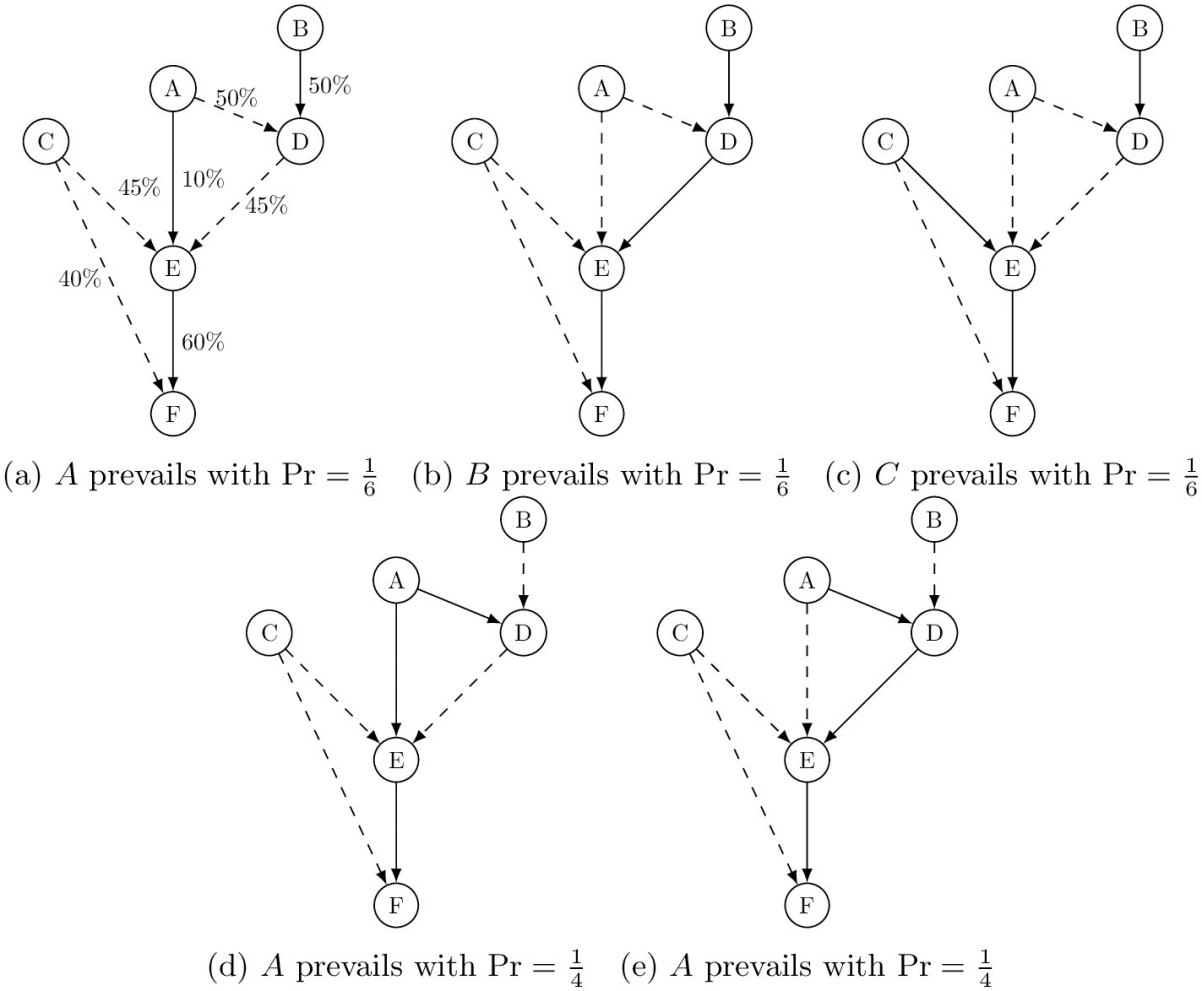
Five possible control paths over *F*.

We continue to use our running example in Fig 1 to develop an intuition. In this stylized network, five control paths (i.e., winning coalitions) are possible that dominate company *F*. The panels in Fig 2 depict these possibilities, where a solid linkage represents a *control* linkage that constitutes the pivot in a majority to dominate in the target company, while a dotted linkage represents a nonzero ownership linkage that fails to achieve controlling ownership.

Suppose that ultimate owner *B* happens to prevail in *D* as shown in the three panels (*a*) through (*c*) in the top row of Fig 2. Under this supposition, none of the ultimate owners (*A*, *B*, or *C*) alone can form a majority coalition over *E* with certainty, so that each ultimate owner has some chance of controlling *E*. For example, if *A* prevails in *E* with probability 1/6 as in Panel (a), the control paths that establish *A*’s control in *F* consist of {*A*, *E*, *F*} and {*B*, *D*}. Alternatively, if *A* happens to prevail in *D* as shown in the bottom panels (*d*) and (*e*) in Fig 2. Then, *A* always control *E* either directly as in panel (d) or indirectly through *D*’s ownership in *E* as in panel (e).

The key question we ask here is how often an intermediate shareholder is indispensable for an ultimate owner to form the ownership structure necessary to control a certain target company. More intuitively, our model of NPF asks how frequently an intermediate shareholder gets on a control path emanating from ultimate owners to target companies. The NPF measures this by calculating two properties of paths that establish control over a target company (i.e., winning coalitions): (1) the probability that each shareholder (including the ultimate owners) can be pivotal in establishing a control path, conditional on the structure of the ownership network and (2) the expected number of target companies over which the said shareholder mediate the ultimate owners’ control. In doing so, we make the following assumptions about voting rights and voting behavior by shareholders.

**Assumption 1 (Transitive voting)**. *If a shareholder j is under the control of another shareholder i, then i always commands j’s decision over its voting rights, and j’s voting decision always aligns i’s without exceptions*.

**Assumption 2 (Voting coordination)**. *All the shareholders j under the control of the same shareholder i always coordinate their voting decisions on a single platform*.

**Assumption 3 (Common shares)**. *Every unit of the ownership shares has the same weight of the voting rights (Although the nature of the rights and specific privileges and restrictions can vary considerably from one company to another, we assume away the non-voting class and veto-power class of the common shares)*.

Consider panels (d) and (e) from the examples above, where *A* prevails in *D*. In each panel a union consisting of *A* and *D* is given by a permutation between them (i.e., {*A*, *D*} and {*D*, *A*}). Thus, there are four possible majority coalitions (or control linkages) vis-à-vis *E*: that is, {*A*, D*}, {*D*, A*}, {*B*, D*, *A*}, and {*B*, A*, *D*}, where a shareholder with an asterisk (*) denotes a pivot in its respective coalition. Namely, both *A* and *D* are pivotal in two of these four possible coalitions, implying that the likelihood that *A* or *D* can be pivotal in a coalition to control *E* is 1/2. Since the probability that *A* controls *D* is also 1/2 (recall that neither *A* nor *B* alone is a majority in *D*), the probability that *A* prevails in *F* is given by $\frac{1}{2}\times \frac{1}{2}=\frac{1}{4}$ in each of the two bottom panels in Fig 2. By the same token, it is straightforward to show that the probability that *A* prevails in *F* is $\frac{1}{6}$ in all the three top panels in Fig 2.

Once we calculate the probability that each shareholder obtains a majority coalition over (i.e., control paths leading to) a target company, we then count the number of target companies that an intermediate shareholder can help an ultimate owner establish control over. For example, in panels (a), (c), and (d), intermediate shareholder *D* is not part of the control path(s) over *F*, meaning that it does not contribute to any of the ultimate owner’s (indirect) control of any other entities downstream thereof, so that it only controls itself. On the other hand, in panels (b) and (e), *D* is on the control paths leading to *F*, in each of which *D* has three companies (including itself) in its downstream.

Taken together, the probability that a control path (i.e., a majority coalition) shown in each of the panels (a) through (c) can be realized is 1/6, and the probability that a control path in panels (d) and (e) can be realized is 1/4. Also, the number of target companies that *D* helps an ultimate owner establish a control path is one in each of the panels (a) through (c), and three both in panels (b) and (e). Thus, the NPF value for *D* is given by the expected number of entities (shareholders or companies) included in a control path that *D* helps to form, which is given by multiplying the probability that each control path is realized and the number of companies included downstream of *D* in each control path:

$$\begin{array}{c} \underset{\text{Panel}(a)}{\underset{︸}{1/6\times 1}}+\underset{\text{Panel}(b)}{\underset{︸}{1/6\times 3}}+\underset{\text{Panel}(c)}{\underset{︸}{1/6\times 1}}+\underset{\text{Panel}(d)}{\underset{︸}{1/4\times 1}}+\underset{\text{Panel}(e)}{\underset{︸}{1/4\times 3}}=11/6. \end{array}$$ (1)

Since the NPF for a shareholder counts the number of the control paths that the shareholder can be pivotal, conditional on the probability that each of those control paths can be realized, it is not straightforward to define “individual” NPF that would capture the one-to-one influence relations that the shareholder might have against a specific target company. In this sense, the concept of the NPF corresponds to the aggregated version of the NPI. We provide in S1 Appendix the formal definition of NPF and NPI as well as the intuition behind these models in a generalized setting.

### Comparing to other models

Before analyzing a global ownership network in the real world, we provide a comparison of the NPF model to related measures proposed in the previous literature. This comparison is intended to illustrate the similarities and differences between our model of NPF and other models that measure “flows” of influence in a network. The most oft-cited measures for the volume of network traffic are PageRank and Betweenness Centrality. Among more recent contributions from the literature on indirect corporate control [5, 6], two studies are relevant to our study: the “power index” proposed by Mercik and Lobos [14] and the “standardized implicit power index” proposed by Karos and Peters [15, 16].

**Fig 3:**
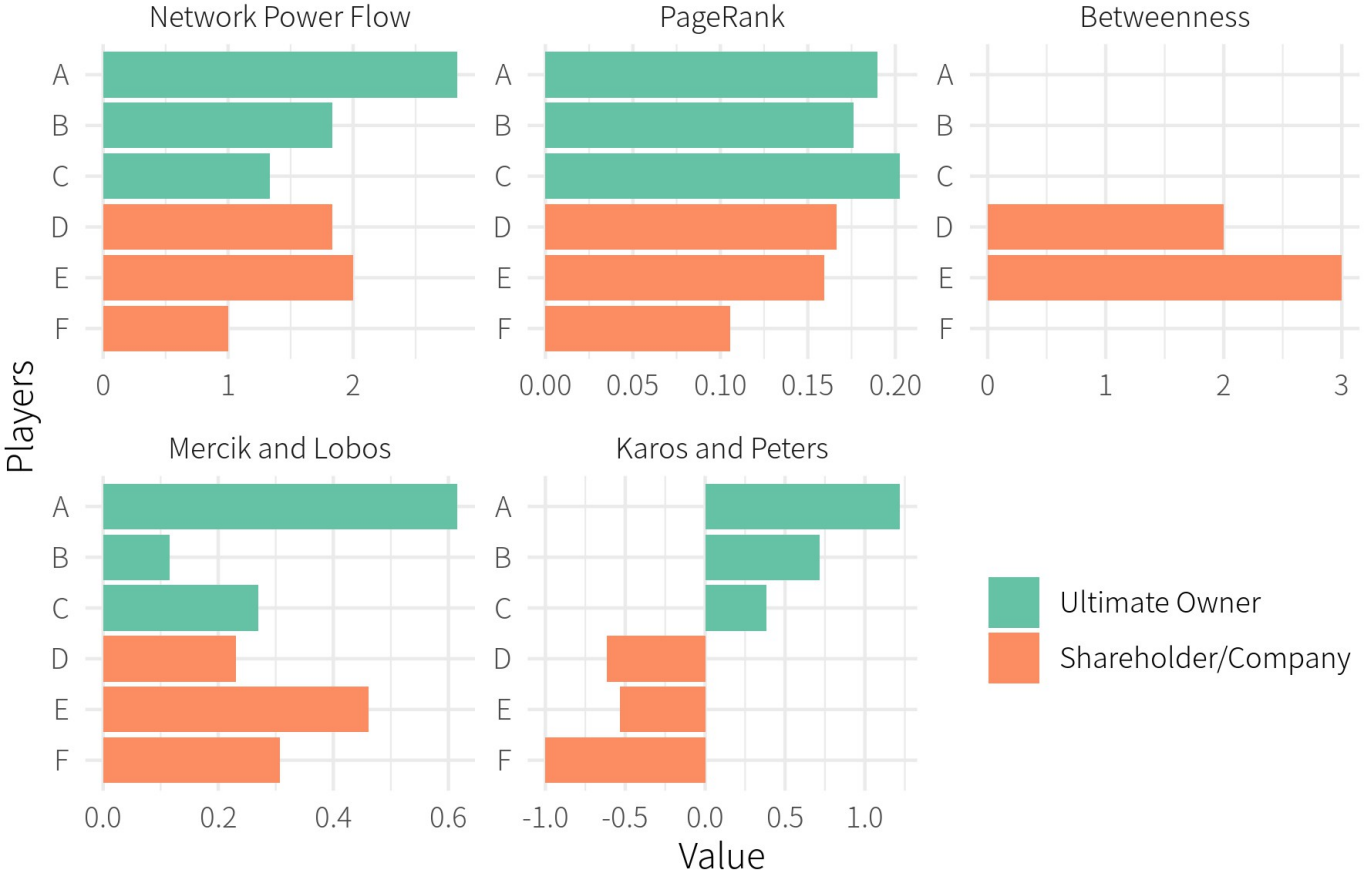
Comparision between NPF and other measurements.

Continuing to use our running example of the ownership network in Fig 1, we apply these four related measures as well as NPF to calculate the relative importance of each entity in the network. The results are summarized in Fig 3, in which we distinguished ultimate owners *A*, *B*, and *C*, from other entities (shareholders and companies), *D*, *E*, and *F*, by colors.

PageRank and Betweenness Centrality (BC hereafter) are expected to differ from NPF since they are not intended to measure the degree of influence in the ownership network. PageRank assigns a larger value to the entities located upstream in the network, weighted by the size of equity stakes held by each entity. To wit, ultimate owner *C* has the largest PageRank value among the ultimate owners with *x*_*CE*_ = 45% and *x*_*CF*_ = 40%; target company *F*, on the other hand, has the smallest PageRank value since it does not own any shares in other companies. BC is intended to measure the amount of information that flows through a node in a network, so it assigns a non-zero value to intermediaries *D* and *E* that bridge entities. As such, the ultimate owners, *A*, *B*, and *C*, as well as target company *F* have the BC value of zero since they are the end nodes.

Unlike PageRank or BC, the “power indices” developed by Mercik and Labos as well as Karos and Peters are intended to measure influence in indirect corporate ownership. Nonetheless, these measures differ from each other. The power index by Mercik and Labos yields a result radically different from NPF; The Karos-Peters power index correlates with the size of the equity ownership but it does not measure how equity stakes are converted to corporate control. Hence, the Karos-Peters index assigns large values to the shareholders upstream in the ownership network (e.g., *A*, *B*, *C*) and small values to those downstream (e.g., *F*).

The power index proposed by Mercik and Labos yields a result that resembles that of NPF but its construction differs in two important ways. First, Mercik and Labos break down a network into two-link connections called *mutual control structure*, and quantify constituting entities’ power to control other entities within each *control structure*. As a result, in our example, the indirect control path from *A* to *F* through *D* and *E* (i.e., Panel (e) in Fig 2) is dismissed. Second, the Mercik-Labos index for intermediate shareholder and companies (i.e., *D*, *E*, and *F*) measures the extent to which they are controlled by others, not the degree they control others. This is the reason for why the Mercik-Labos power index is smaller for *D* than for *F*. Yet, *F* cannot control any entities other than itself, implying no power to control the market. Therefore, the Mercik-Labos power index is not adequate for measuring the contribution of intermediary shareholders to the indirect control established by ultimate owners.

In contrast, NPF successfully quantifies the amount of influence that a shareholder has over the flow of control held by an ultimate owner. As such, any given shareholder’s NPF value does not necessarily correlate with the amount of equity stake held by the shareholder. As shown in Fig 3, the ultimate owners are not always influential. For example, the NPF values for ultimate owners *B* and *C* are less than those for intermediaries *D* and *E*. The intermediate shareholders *D* and *E* have large NPF values because they are the critical “hubs” in the control paths, such that a large amount of corporate control “flows” through these nodes. In particular, Company *D* has the highest value among intermediaries since it is an indispensable hub for two coalitions that control *F* (i.e., Panel (c) and (e) in Fig 2). In contrast, the NPF of *F* is the lowest since it has no downstream companies. This aspect of NPF coincides with, and reflects, the Betweenness Centrality notion in terms of the amount of influence over the flow of corporate control.

## Data and method

### Data

We use *Bureau van Dijk*’s Orbis database on the publicly listed companies and their shareholders, as of December 1, 2020, to analyze the global ownership network [17]. We obtained the data in early January 2021. The ownership network that we analyze represents the *global* network in the sense that, unlike previous studies [15, 18–26], our analysis includes companies and shareholders irrespective of their registered country or sectors. We also include in our analysis the shares in *any* publicly listed company, unlike previous studies focusing only on transnational corporations [4], large companies [27], or state-owned enterprises [28]. Nor do we exclude individuals or governments in the analysis of the shareholders who own equity shares in companies in global economy. Further, we exploit the unique identification number provided by the Orbis database for each shareholder to determine whether separate institutional investors should be deemed as a single investing entity if they are owned by the same individual. We construct a directional graph representing the global ownership network that consists of 146,221,188 *nodes* representing companies *j* and their shareholders *i*, of which 78,692,111 are ultimate owners, as well as 105,555,741 *edges* representing ownership ties *x*_*ij*_ between them.

However, since the Orbis database contains a multitude of minuscule companies that are only owned by their respective owners and hence are not connected to the global network, we drop those observations and limit our analysis to the giant connected components (GCC) including global entities such as the national governments of major powers as well as the world’s largest financial institutions. The resulting GCC as the data for the global ownership network consists of 16,897,741 nodes (i.e., the *i*’s and the *j*’s), of which 7,955,320 are ultimate owners, and 21,300,018 ownership linkages *x*_*ij*_ between them.

Limiting our analysis to the GCC does not lose generality because small companies outside of the GCC are not connected to the global ownership network. Further, as we shall demonstrate shortly, the NPI and NPF values for a small company outside of the GCC only reflect the power to control its own company, so that this exclusion rule should not affect the structure of corporate control in the global network. As part of the robustness analysis, we also run our analysis on the weakly connected network and the results remain unchanged.

### Algorithm

To apply our model to the real-world data, we develop an algorithm to calculate NPF for each shareholder by modifying the algorithm to calculate NPI that we have proposed elsewhere [7]. The exact calculation of these quantities is theoretically possible but computationally demanding in practice. This is especially true when applying our model to a large network containing millions of entities.

Our algorithm displayed here (in Algorithm 1) is a numerical approximation of NPFs, based on random sampling (the code that implements this algorithm in Python is available in https://doi.org/10.5281/zenodo.8105817). The idea for our method is simple. Suppose a control path from ultimate owner *i* towards a target company *j*, mediated by an intermediary *k*. Then, by sequentially finding a pivot given the structure of control paths in the upstream for each *k*, we obtain one of the possible control paths, (*N*, *Y*), and an indirect control matrix, $\bar{Y}={\left(I-d\cdot Y\right)}^{-1}$. Therefore, letting ${\bar{y}}_{ij}^{\left(t\right)}$ denote the element of $\bar{Y}$ in the *t*-th iteration, we can rewrite the estimator of NPF as ${\hat{p}}_{ij}=\sum_{t=1}^{T}{\bar{y}}_{ij}^{\left(t\right)}v_{j}/T$. Interested readers should refer to [7, 29] for discussion of the relations between calculation errors and the number of iterations as well as the modification of the algorithm to deal with the potential problem of initial values.

**Algorithm 1** An algorithm to calculate NPF

**Input**: A set of entities, *N* = {1, …, *n*}; a matrix of voting rights *x*_*ij*_ ∈ [0, 1]; a vector of quotas *q*_*j*_ ∈ [1/2, 1]; a vector of values $v_{j}\in R$; # of iterations, T∈Z; a damping factor, *d* ∈ (0, 1]; an inflation factor, *ρ* ∈ (0, 1]

**Output**: NPF, $\hat{p}\in R^{n}$

$L_{i}^{D}\leftarrow i,L_{i}^{I}\leftarrow i$ ▹ Initialize the labels for direct and indirect control

 **for** *t* ∈ {1, 2, …, *T*} **do**

  **for** *j* ∈ *N* **do**

$U_{j}^{i}\leftarrow$ a random sample of $\left\{k|L_{k}^{I}=i\right\}$    ▹ Create unions of *i*

   *N*_*j*_← a random sample of $U_{j}^{i}$        ▹ Shuffle unions

   *x*_*j*_ ← 0      ▹ Initialize the sum of voting shares

   **while** *x*_*j*_ ≤ *q*_*j*_ **do**       ▹ Find a pivot and an indirect owner

$L_{j}^{D}\leftarrow k,L_{j}^{I}\leftarrow L_{k}^{I}$

    *x*_*j*_ ← *x*_*j*_ + *x*_*kj*_

   **end while**

  **end for**

  **for** (*i*, *j*) ∈ *N* × *N* **do**      ▹ Create a control network *Y*

   **if** $L_{j}^{D}=i$ **then**

    *y*_*ij*_ ← 1

   **else**

    *y*_*ij*_ ← 0

   **end if**

  **end for**

${\bar{y}}^{\left(t\right)}\leftarrow {\left(I-d\cdot Y\right)}^{-1}v$ ▹ Calculte the downstream values

 **end for**

$\hat{p}\leftarrow \sum_{t=1}^{T}p^{\left(t\right)}/T$ ▹ Calculate NPFs

$\hat{p}\leftarrow \hat{p}/\rho$

This algorithm yields a consistent estimator of NPF if there is no cross-ownership causing loops in the network. That is, as the number of iterations increases, the estimate approaches the true parameters (Proposition 3 in S1 Appendix). If there is cross-ownership between *i* and *j*, the algorithm cannot determine which entity is the ultimate owner, so that a loop occurs in counting ${\bar{y}}_{ij}^{\left(t\right)}$. This indeterminacy is terminated by randomly determining *i* or *j* is the ultimate owner. Since the loop that occurs in the process of assigning the controlling power to *i* and *j* in cross-ownership will inevitably inflate the NPF values, we introduce a damping factor *d* to correct for the inflation. Following the original PageRank algorithm, we set *d* to 0.85 [30]. That is, for each unit of separation in the ownership linkage *x*_*ij*_, the estimated NPF value will be discounted by *d* = 0.85.

The last step in the algorithm is to divide the estimated values of NPF by an “inflation factor” *ρ* ∈ (0, 1]. Since the damping factor significantly underestimates the NPF values for the shareholders *i* if *i* is separated from a target company *j* with many linkages further down in the network, the inflation factor *ρ* corrects for underestimation so that the NPF values are comparable to the corresponding values of NPI. The exact value of *ρ* is determined by actual data in the next section.

## Analysis

We calculate NPI and NPF values for each shareholder *i* both at the “individual” and “aggregate” levels. An individual NPI measures the power of control possessed by *i* against each specific target company *j* when *i* is an ultimate owner. Similarly, the “aggregated” NPI and NPF values sum up the individual-level quantities at the systemic-level of the ownership network for each *i*. Following our previous study on NPI [7], our approximation algorithm iterates the calculation for *T* = 20, 000 times with the damping factor set at *d* = 0.85 and the quota set at *q*_*j*_ = 0.50.

A caveat is in order regarding an additional assumption that enables us to analyze the data. A major limitation of the *Orbis* database is its limited coverage [31]. When all the shareholders and their amount of shareholding are not recorded for any given company in the GCC (Giant Connected Component), we assume that those missing (or unknown) shareholders cast their vote(s) in line with the management in calculating NPI and NPF values. In making this assumption, we follow the seminal study on share ownership and voting power by Berke and Means in 1932, since most of missing information in the *Orbis* database is for the dispersed share(s) less than 1%, which are the focus of the Berke and Meads study [32].

### Contrasting NPF to NPI in the data

**Fig 4:**
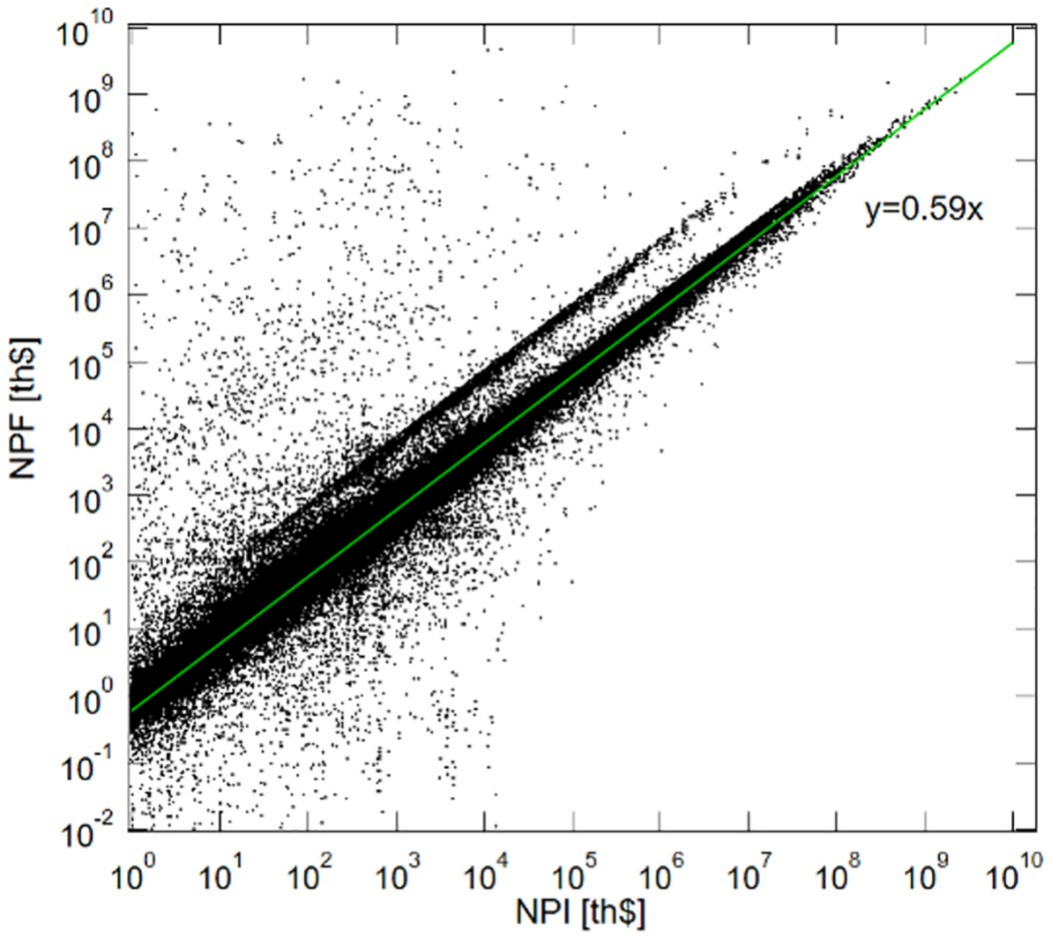
Comparing NPF to NPI.

We begin our analysis by comparing NPF to NPI at the aggregate level. Fig 4 plots the logged values of NPI and NPF at the aggregate level, both of which are weighted by sales of the target companies in U.S. trillion dollars *v*_*j*_*p*_*i*_ = ∑_*j*_ *v*_*j*_*p*_*ij*_. While only the ultimate owners have NPI values greater than zero (> 0), our algorithm assigns nonzero NPI values to intermediate owners as well unless the ultimate owner is identifiable due to cross-ownership.

This figure also includes the OLS fitted curve, for which the coefficient turns out to be 0.59. As we noted above in the algorithm section, the NPF values are underestimated compared to the corresponding NPI values because of the damping factor *d* to adjust for overshoot of power typically due to cross-ownership in the data. The OLS coefficient of 0.59 measures the extent to which the *d* underestimates the NPF values on average.

The vast majority of shareholders are plotted around the OLS curve and their NPF values are slightly less than the corresponding NPI values. Recall that the damping factor *d* discounts a shareholder’s NPF value each time its influence extends to a unit edge-length downstream in a control path. A large discount is expected if the shareholder holds very large equity stakes under management going through the network.

A cluster of shareholders that forms a parallel line just above the OLS curve have their NPF values generally greater than their corresponding NPI values, despite the damping factor *d*. The fact that NPF values are greater than NPI values suggests that these shareholders’ corporate control may be extended downstream in the network and hence their influence over the flow of corporate control is amplified perhaps because of cross-ownership.

The shareholder observations that are plotted well above the OLS curves have their NPF values much greater than the NPI values. Our algorithm yields substantial differences between NPF and NPI values if the algorithm recounts the target companies repeatedly as the control power flows through these entities multiple times in a circle. We interpret these observations as evidence that extensive cross-ownership does exist in our dataset.

In what follows, for the ease of comparison, we report the adjusted values of NPF, which are divided by *ρ* = 0.59 (i.e., the OLS coefficient). We do this because, at the conceptual level, NPF should be interpreted as a generalized version of aggregated NPI and as a generic measure of the power of corporate control for any shareholder the ultimate owners and intermediaries alike.

### Detecting cross ownership

Another useful way to use NPF in comparison to NPI is to detect circular shareholding due to cross-ownership in the data. Note that the estimated values of NPF and NPI for the “influential” ultimate owners with large NPI values converge to the linear regression curve as they approach their maximum values, as is shown in the upper right corner of Fig 4. Consequently, the adjusted NPF values for the shareholders with the largest NPF values listed in Table 1 are expected to be roughly equal to their corresponding NPI values if shareholder *i* is an ultimate owner—i.e., unless NPI = 0 or NPI ≃0.00 for *i*.

**Table 1:** Ownership structure of influential shareholders (Top 15 NPF).

	Shareholder *i*	NPF^(1)^	NPI^(2)^	HHI^(3)^	NPI_*ji*_ ^(4)^	Largest Owner *j*
1	Government of China	16.75	17.03	0	1	= *i*
2	SASAC^(5)^	11.91	0	1	1	Government of China
3	BlackRock	7.87	0.00	0.05	0.14	Government of Kuwait
4	Vanguard Group	7.73	0.00	0.20	0.21	Vanguard Index Funds
5	Sinopec Group	4.05	0	1	1	Government of China
6	State Street Corp.	3.62	0.00	0.03	0.08	Johnson Family
7	Capital Group Co.	2.89	2.80	0	1	= *i*
8	Fedelity MR	2.81	0.00	1	1	Johnson Family
9	Gov’t of Norway	2.74	2.57	0	1	= *i*
10	JPMorgan Chase	2.61	0.00	0.03	0.10	Capital Group Co.
11	BPCE SA	2.50	0.39	0.05	0.50	= *i*
12	China Petroleum Corp.	2.20	0.00	1	1	Government of China
13	Master Trust Bank of Japan	2.02	1.76	0	1	= *i*
14	Government of Russia	1.88	1.94	0	1	= *i*
15	Johnson Family	1.77	2.25	0	1	= *i*

*Note*:

^(1)^ NPF: Aggregated NPF for *i* weighted by operating revenue in U.S.$tn.

^(2)^ NPI: Aggregated NPI for *i* weighted by operating revenue in U.S.$tn.

^(3)^ HHI: Herfindahl–Hirschman index

^(4)^ NPI_*ji*_: Individual NPI over *i* held by the largest ultimate owner *j*

^(5)^ SASAC: State-owned Assets Supervision and Administration Commission

An exception to this rule in Table 1 is BPCE SA whose NPF value is substantially larger than its NPI. One of the reasons for the inflated NPF values is cross-ownership as it causes recursive counting of corporate control in computing the NPF values with our algorithm.

Cross-ownership exists when two or more companies own shares in one another. BPCE SA is the parent company of a French banking network, holding 100% ownership in two major banking groups, the Banque Populaire (BP) and the Caisse d’Epargne (CE). While the Orbis database correctly indicates that BPCE SA itself is one of the ultimate owners of itself, each of its subsidiary groups is also an ultimate owner of its parent company BPCE (albeit with a small share), which results in cross-ownership. Consequently, our algorithm repeatedly recounts BPCE’s control of the BP subsidiaries and CE subsidiaries and its NPF value turns out to be substantially greater than its NPI value despite the damping factor *d*.

Since NPF can be thought of as a generalized version of NPI, and our algorithm makes NPF and NPI comparable especially for the shareholders with large corporate influence, the outliers among the influential shareholders are likely to indicate excessive cross-ownership.

### Who has control? Governments and the “Big Three” asset managers

Among the top 15 influential shareholders with the largest NPF values listed in Table 1 are either governments, state-owned enterprises or American financial institutions. This pattern is pervasive at least among the top 1,000 shareholders. Among the 15 top shareholders, three are state governments (i.e., the People’s Republic of China, Norway, and the Russian Federation). In addition, the Republic of Korea ranks 28th, France ranks 32nd, and Singapore ranks 62nd. This result is consistent with our previous study on NPI based on the Orbis database of 2016, where 4 out of 15 shareholders with the largest NPI values were also state governments (see Table 2 in [7]).

The second largest shareholder is SASAC (i.e., State-owned Assets Supervision and Administration Commission) that is part of the executive branch of the Chinese government. Since it is fully owned (and the Orbis database indicates its shares are fully owned) by the government, we can assume that SASAC’s NPF is subsumed by the NPF (or equivalently the NPI) held by the Chinese government. If so, this makes BlackRock effectively the second largest shareholder in the world in its capacity to control corporations. At the same time, this highlights the Chinese government’s uniquely strong power of corporate control around the world, as its aggregated NPF value is more than double of BlackRock’s. This result is consistent with a previous study emphasizing the role of state-owned enterprises [28].

Financial institutions also have a tremendous capacity to control corporations around the world. In particular, the so-called “Big Three” asset managers (i.e., BlackRock, Vanguard, and State Street) stand out in terms of their NPF values. Moreover, since Sinopec Group and China Petroleum are China’s state-owned companies managed by SASAC, their power of corporate control (i.e., their NPF values) are presumably subsumed by the Chinese government’s NPF in the same manner as SASAC. This means that BlackRock and Vanguard, with roughly same NPF values, are effectively the second largest players in the global ownership network with a wide margin to the next tier that includes State Street and Capital Group with NPF values that are less than half of BlackRock’s or Vanguard’s.

Possession of power of corporate control in terms of NPF, however, does not necessarily imply that shareholders can or do exercise their influence over their target companies. Depending on the structure of ownership around it, a shareholder may not be able to use its own voice. We turn to this issue in the next section.

### Ownership structure and independence

Not all the shareholders with great power of corporate control are the same. There are two types of intermediate shareholders with high NPF values in terms of their independence: Those that can exercise their power of corporate control free of an external influence and others that cannot voice their own will. To measure the degree of autonomy a shareholder *i* possesses in exercising its power of corporate control, we introduce a quantity adopted from the Herfindahl-Hirschman Index (HHI), which was originally intended to measure the degree of monopoly or diversity in a given industry [33]. Here, we think of the total share of a target company *i* as a single market, and quantify the degree of *j*’s monopoly of ownership share in *i* among its ultimate owners as follows:

$$\begin{array}{c} {\text{HHI}}_{i}=\sum_{j\neq i}{\text{NPI}}_{j\to i}^{2}. \end{array}$$ (2)

Our version of the Herfindahl-Hirschman Index over *i*, denoted by HHI_*i*_, measures how dispersed the ultimate owners’ *j* influences are over a target company *i*, as a function of the *individual* NPI value held by *j* over *i*. The HHI_*i*_ values ranges from 0 to 1, where HHI_*i*_ = 0 indicates that *i* is fully independent, or not owned by any entity, while HHI_*i*_ = 1 indicates that a single ultimate owner *j* possesses dominant control over *i*. As the HHI_*i*_ value gets smaller, the shares in *j* are more fragmented and widely held, so that *i* becomes more autonomous.

By comparing NPI, HHI_*i*_, and NPI_*ji*_ in Table 1, we can categorize the shareholders into several types. The first category is an obvious one, which is the ultimate owners themselves who have nonzero NPI values and HHI_*i*_ = 0. Since the ultimate owner of any ultimate owner is itself (*j* = *i*) by definition, its NPI_*ji*_ value over itself is one (1). The ultimate owners are fully independent of any external influence in exercising the power of corporate control. The examples from Table 1 include governments (China, Norway, Russia), financial institutions (Capital Group, Master Trust Bank of Japan), and the Johnson Family (the owner of Fedelity).

A second type of shareholder also enjoys autonomous decision-making power free of external ownership influence, as in the case where HHI_*i*_ is sufficiently low but the largest ultimate owner *j* does not have a dominant share so that *NPI*_*ji*_ is also low. A typical example is a widely-held company such as BlackRock with HHI_*i*_ = 0.05 and its ultimate owner’s (the Kuwaiti government) share is small enough that the probability that it can establish dominance in BlackRock is given by NPI_*ji*_ = 0.136. These widely-held companies and large financial institutions turn themselves into power houses by collecting a massive amount of equity stakes that carry zero to minuscule voting power from fragmented (often individual) investors who are motivated not by corporate control but by economic returns. They amass voting power on behalf of their investors without being influenced by the investors.

The Vanguard Group also is independent of any outside corporate influence because it is not publicly owned; rather it is privately owned by five of its own funds with a roughly equal share each, so that HHI_*i*_ ≃ 0.201 and its largest owner has individual NPI_*ji*_ ≃ 0.213. This unique ownership structure allows Vanguard Group to maintain independence from any entity’s influence in the global ownership market. Each of these Vanguard funds collects money from (individual) investors, which the Vanguard Group leverages to purchase ownership of companies around the world, in the same fashion as other large financial institutions. S2 Appendix provides more detail on the ownership of Vanguard Group and BlackRock.

The last and third category of shareholders are state-owned enterprises or any family-run businesses including the Walton family running Walmart and the Johnson family controlling Fedelity MR, where HHI_*i*_ = 1 and NPI_*ji*_ = 1. These shareholders possess great power of corporate control but cannot exercise that power for their own sake: they are subordinate to their single ultimate owner. In these cases, the high NPF value for the subsidiaries is derived from their owner’s NPI. The shareholders of this type represent the will of their owners in exercising their power. Ultimate owners with this type of intermediate shareholders are less likely to suffer from moral-hazard problems. SASAC is a good example, where it has enormous power of corporate control but that power would not be exercised independently of the Chinese government’s will.

#### Government control: The case of the U.S., China, and Russia

If a shareholder is powerful (in terms of its NPF) but under the influence of its owner(s), the shareholder itself may not be able to exercise that power because its owner’s power (i.e., NPI) runs through the shareholder who happens to be on the owner’s control path. The implications of such power relations are not negligible if the controlling owners in question are political entities. For example, Sinopec Group and State Street Corp have roughly equal amounts of influence over the power of corporate control that flows through them as indicated by NPF in Table 1. However, acquiring control over Sinopec Group is not feasible since it is fully owned by the Chinese government.

To explore the role of governments in corporate control, we compare three politically salient states in world politics: The United States, the People’s Republic of China, and the Federation of Russia. Tables 2–4 respectively list the shareholders with the largest NPF values in each country.

**Table 2:** American shareholders with top 10 NPF values in December 2020.

	Shareholder *i*	NPF	HHI	NPI_*ji*_	Largest ultimate owner *j*
1	BlackRock, Inc	7,870.31	0.051	0.136	Government of Kuwait
2	Vanguard Group Inc.	7,730.05	0.201	0.213	Vanguard Index Fund
3	State Street Corp.	3,617.27	0.027	0.078	Johnson Family
4	Capital Group Co.	2,887.78	0	1	= *i*
5	FMR LLC	2,806.97	1	1	Johnson Family
6	JPMorgan Chase & Co.	2,614.27	0.029	0.095	Capital Group Co.
7	T. Rowe Price Group Inc.	1,528.29	0.025	0.055	Capital Group Co.
8	Bank of America Corp.	1,389.47	0.035	0.108	Warren Buffett
9	Berkshire Hathaway Inc.	1,369.42	0.249	0.490	Warren Buffett
10	Vanguard Bond Index Funds	1,295.00	0	1	= *i*

*Note*:

NPF: Aggregated NPF for *i* weighted by operating revenue in U.S.$bn.

NPI_*ji*_: Individual NPI over *i* held by the largest ultimate owner *j*.

**Table 3:** Chinese shareholders with top 10 NPF values in December 2020.

	Shareholder *i*	NPF	HHI	NPI_*ji*_	Largest ultimate owner *j*
1	Government of PRC	16,752.72	0	1	= *i*
2	SASAC	11,909.08	1	1	Government of PRC
3	Sinopec Group	4,049.00	1	1	Government of PRC
4	China Petroleum	2,198.80	1	1	Government of PRC
5	China Petroleum & Chemical	1,649.12	1	1	Government of PRC
6	China National Petroleum	1,590.45	1	1	Government of PRC
7	State Grid Corp. of China	1,412.59	1	1	Government of PRC
8	Ministry of Finance of PRC	1,098.76	1	1	Government of PRC
9	Shanghai SASAC	1,011.38	1	1	Government of PRC
10	Shanghai Municipal People’s Government	859.68	1	1	Government of PRC

*Note*:

NPF: Aggregated NPF for *i* weighted by operating revenue in U.S.$bn.

NPI_*ji*_: Individual NPI over *i* held by the Chinese government *j*

**Table 4:** Russian shareholders with top 10 NPF values in December 2020.

	Shareholder *i*	NPF	HHI	NPI_*ji*_	Largest ultimate owner *j*
1	Government of the Russian Federation	1,877.07	0	1	= *i*
2	VTB Capital	1,158.54	1	1	Government of Russia
3	VTB Capital IB Holding	986.27	1	1	Government of Russia
4	Federal Agency for State Property Management	966.38	1	1	Government of Russia
5	VTB Capital Holding	838.55	1	1	Government of Russia
6	VTB Bank	785.71	1	1	Government of Russia
7	Gazprom	621.84	1	1	Government of Russia
8	Rosneft Oil Co.	496.74	0.21	0.37	Government of Russia
9	Lukoil Oil Co.	370.51	1	1	Government of Russia
10	Rosneftegaz	291.10	1	1	Government of Russia

*Note*:

NPF: Aggregated NPF for *i* weighted by operating revenue in U.S.$bn.

NPI_*ji*_: Individual NPI over *i* held by the Russian government *j*

Table 2 shows that none of the most influential shareholders registered in the United States are controlled by the U.S. government. Also, most of them are widely owned except for the privately owned corporations (including Capital Group, Fedelity, and Berkshire Hathaway) with financial institutions or individuals as their ultimate owners. The Government of Kuwait is the largest owner of BlackRock, but it is far from holding a dominant share.

In contrast, as shown in Table 3, all the corporations or shareholders with top-15 NPF values in China are fully owned and controlled by the Chinese government. As such, the Government has the largest NPF value, followed by SASAC. This is as expected since the Chinese government is formed by a communist party.

The situation in Russia is the same as in China, except that its influence in the global ownership market is an order of magnitude smaller than the Chinese influence measured with the aggregated NPF values. Also note that all the companies listed here belong to the so-called “Russian Oligarchs” who later became subject to economic sanctions by the Western democracies in the wake of the Russian invasion of Ukraine in 2022. Although the Russian government’s ownership in Roseneft Oil company falls short of controlling ownership, the company is still a state-owned company since the majority of the shares are held by Rosneftgaz (40.4%) and Russia’s National Settlement Depository (10.43%) and the controlling shareholder in each is the Russian government.

#### Is NPF only useful for dispersed ownership?

Since NPF is particularly useful in detecting the formation of a majority through coalescing dispersed ownership, one may wonder if NPF (or NPI for the same token) might be less relevant for China or Russia where holding a majority interest is believed to be a common practice among state-owned investors. To examine if this common belief is the case, we calculate the NPF values over domestic companies and foreign companies separately. Table 5 reports the top 10 NPF values for “transnational ownership” and for “domestic ownership.” Ownership is domestic if *i* and *j* reside in the same country or transnational otherwise, where a shareholder *i* holds ownership interest directly in *j* (i.e., *i* → *j*) or indirectly in *j* through its ownership in *k* (i.e., *i* → *k* → *j*).

**Table 5:** Domestic & transnational NPF values: Top 10 in December 2020.

	Transnational Ownership			Domestic Ownership		
	Shareholder *i*	NPF	*φ*	Shareholder *i*	NPF	*φ*
1	BlackRock	4.64	0.59	Government of China	15.41	0.92
2	Vanguard Group	4.08	0.53	SASAC	10.99	0.92
3	Government of Norway	2.60	0.95	Vanguard Group	3.65	0.47
4	BPCE SA	1.83	0.73	Sinopec Group	3.49	0.86
5	Capital Group Co.	1.74	0.60	BlackRock	3.23	0.41
6	State Street Corp.	1.72	0.47	China Petroleum Corp.	1.92	0.88
7	JPMorgan Chase	1.59	0.61	State Street Corp.	1.90	0.53
8	Fedelity MR	1.50	0.54	Government of Russia	1.74	0.93
9	Vanguard Index Funds	1.38	0.99	Master Trust Bank of Japan	1.48	0.73
10	Government of China	1.34	0.08	China National Petroleum Corp.	1.46	0.92

*Note*:

NPF: Aggregated NPF for *i* weighted by operating revenue in U.S.$bn.

*φ*: Ratio of transnational or domestic NPF to total NPF

Except for Vanguard Index Funds, all the shareholders with top 10 “domestic” or “transnational” NPF also appear in the list of the shareholders with top 15 overall NPF values reported in Table 1. This result suggests that large-scale ownership influence is not confined to a single country in the global ownership network. This is also the case for “statist” economies like China, where a central government retains a substantial control over domestic economy.

Although the ownership influence possessed by the government of China and its state-owned enterprises predominantly is over companies in China (≈ 92%), its influence over the control of foreign companies is also substantially large as its transnational NPF value is ranked 10th, despite the small portion in their portfolio (≈ 8%). This result suggests that NPF is instrumental in describing how otherwise predominantly domestic ownership may infiltrates outward through the web of the shareholding network and gains the potential control in widely held companies abroad. The fact that the Russian government has a very small “transnational” NPF values suggests that Russia’s state-owned influential shareholders listed in Table 4 may not have ownership interest in companies abroad.

In sum, NPF (and NPI by extention) proves useful in describging the formation of control over widely held companies that can be vulnerable to the formation of “hidden control” through the web of *indirect* corporate ownership. In this sense, what matters for NPF and NPI is not whether an investor hold a diversified portofolio, but whether a target company’s onwership is fragmented since dispersed ownership may create a room for shareholders to play “weighted voting games” in the ownership network.

### Converting equity stakes to corporate control: Comparing PageRank to NPF

A common way to measure the importance of a node in terms of the volume of traffic flow in a network is PageRank. In this study, we interpret PageRank as measuring the volume of equity stakes that flow through a shareholder to the target companies downstream in the network. A theoretical comparison between PageRank and NPF has been provided above. This section shows that their empirical comparison helps us understand how a shareholder’s equity stakes might be converted into the power to control target companies.

Table 6 lists the shareholders whose PR values are ranked in the top 10 as well as the governments of the Russia Federation and the United States of America as reference points. To make it comparable to NPF, the PageRank value of ownership shares is also weighted by the target company’s operating revenue in trillion U.S. dollars as well as by 0.59^−1^ (i.e., the OLS coefficient of NPF on NPI found in Fig 4).

**Table 6:** Shareholders with top 10 PageRank (PR) values in December 2020.

	Shareholders	PR^(1)^	NPF^(2)^	NPF/PR
1	Government of China	14.96	16.75	**1.12**
2	SASAC	10.18	11.91	**1.17**
3	Vanguard Group	8.93	7.73	0.87
4	BlackRock	8.26	7.87	0.95
5	State Street Corporation	3.86	3.62	0.94
6	Vanguard Index Funds	3.79	1.39	0.37
7	Government of Norway	3.32	2.74	0.82
8	Capital Group	3.24	2.89	0.89
8	Fedelity MR	3.19	2.81	0.88
10	JPMorgan Chase & Co	2.83	2.61	0.92
…				
16	Government of the Russia Federation	1.62	1.88	**1.16**
…				
464	Government of the Unites States of America	0.19	0.19	**1.00**

*Note*:

^(1)^ PR: PageRank score weighted by operating revenue in trillion U.S. dollars

^(2)^ NPF: Aggregated NPF values weighted by operating revenue in trillion U.S. dollars

These shareholders with the large traffic volume of equity stakes are governments and American financial institutions BlackRock, Vangaurd, and State Street (i.e., the “Big Three” asset managers). Although previous studies emphasize how the large financial institutions dominate the global capital market, our result reveals that they are actually small relative to political entities, suggesting that these previous claims about financial institutions are overstated [3, 4]. In terms of PageRank values, the Chinese government is the largest shareholder (PR = 14.96) with a wide margin to the second. As noted above, since SASAC is part of the Chinese government, it is safe to assume that SASAC’s PR value is subsumed by that of the PRC government.

Similarly, the NPF value for the Chinese government alone is more than twice as large as that of BlackRock or Vanguard or the combination of the two. The NPF of China’s SASAC alone is 50% greater than that of BlackRock, meaning that SASAC enjoys much greater influence over the power of control that flows through it than does the most influential asset manager. SASAC is arguably the most powerful asset manager in the world.

The third column in Table 6 shows the ratio between NPF and PageRank which we interpret as measuring how effectively each shareholder converts its equity shares into a controlling interest in companies in the network. If this ratio is less than one, the shareholder’s investing portfolio is less effective in this conversion. If greater than one, the shareholder is effective in obtaining corporate control based on the equity stakes it controls over the target companies.

An interesting pattern is that, among the shareholders listed in this table, the ones with an NPF/PR ratio greater than or equal to one are the governments of sovereign states except for Norway; and all other shareholders with rations less than one are large financial institutions. This make sense since financial institutions presumably invest in equity shares for economics returns; their diversified equity investments are unlikely to be concentrated on particular target companies to establish controlling interest in those companies. In other words, if a shareholder has an NPF/PR ratio greater than one, we speculate that the shareholder has a motive other than economic returns for its shareholding since this indicates that their investing portfolios are skewed and concentrated on a small set of companies. This speculation is corroborated by the fact that companies with numerous subsidiaries (such as holding companies) tend to have large values for this ratio: for instance, China Petroleum Corporations with a ratio of 5.08, Plains All American GP (4.37), and RN-NeftKapitalInvest (4.13), which is a subsidiary of Russian’s oil firm Rosneft.

Given this, it is interesting to see that the NPF/PR ratio for the government of Norway is roughly the same with other asset managers. Other countries with a ratio of less than one include South Korea (0.93), Kuwait (0.92), and Singapore (0.86) among those with high PageRank values. In contrast, the Chinese and Russian governments are incredibly effective in converting their equity assets into controlling ownership in their target companies. In addition, the governments of large economies with a tradition of state-owned enterprises also tend to have high NPF values relative to their PageRank such as France (1.11) and Japan (1.17).

**Fig 5:**
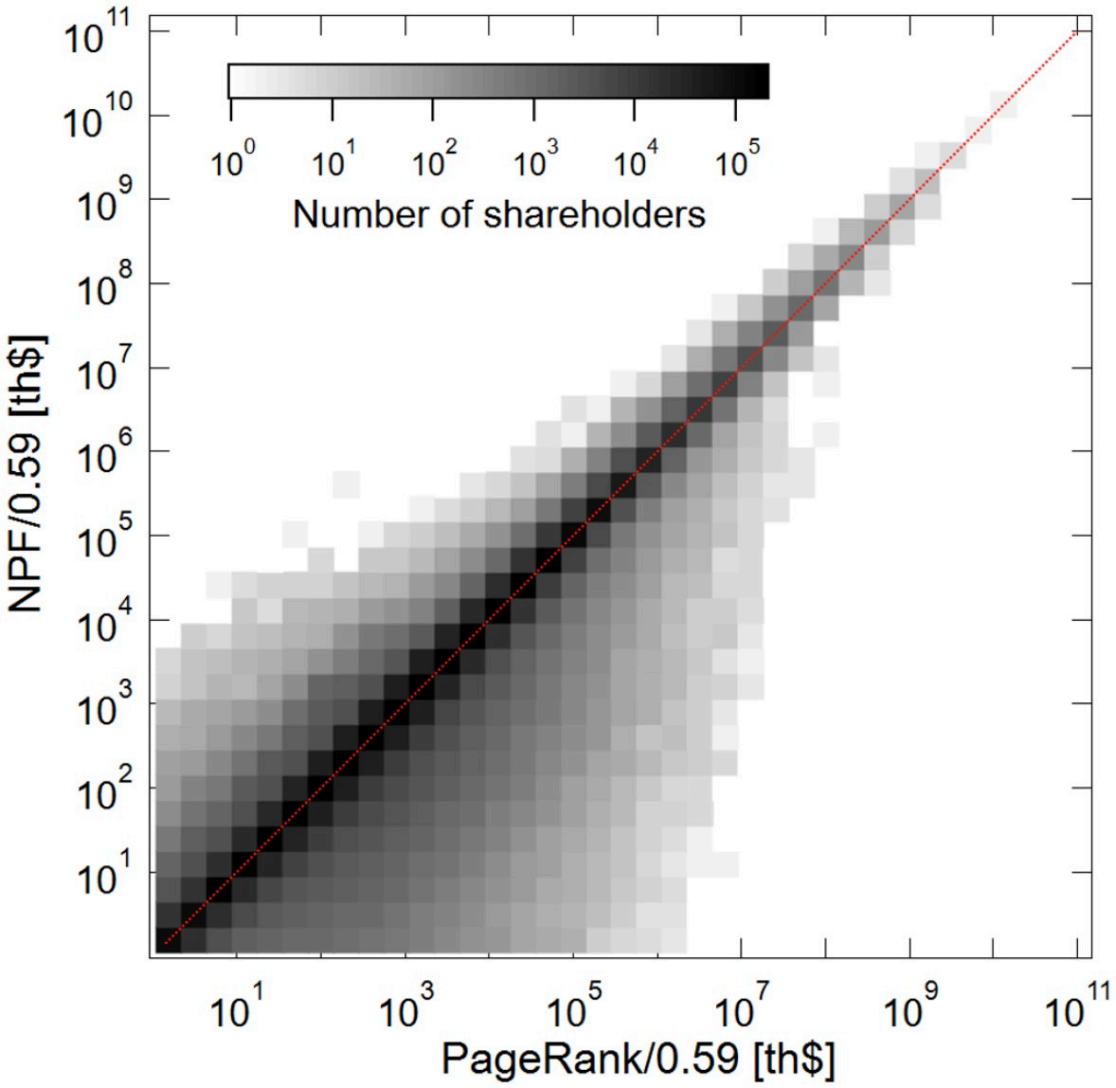
Comparing NPF to PageRank.

The heat-map in Fig 5 depicts the overall relationship between PR and NPF, where the 45-degree line represents an NPF/PR ratio of one. We note two observations. First, shareholders are highly concentrated around the 45-degree line for any size of PageRank or NPF, meaning that many NPF/PR ratios are around 1.0. Second, the vast majority of shareholders are located below the 45-degree line rather than above it, so the the norm is for this ratio to be less than one. This suggests that most of the shareholders in the network possess relatively little power of corporate control relative to the volume of their equity stakes over the companies located downstream in the ownership network. Their ownership of equity shares is less likely to contribute to the possession of the power of corporate control. This overall norm underscores the peculiarity of the tendency of national governments to have substantial power of corporate control relative to the equity stakes that they have under their influence.

## Conclusion

The objective of this paper has been to propose a new model and algorithm, called Network Power Flow (NPF), to measure the amount of influence a shareholder has over the flow of corporate control in the global shareholding network. The immediate motivation for this was to address the problem that our previous work on Network Power Index (NPI) does not adequately describe ownership influence of the publicly held companies, such as “Big Three” asset managers in spite of their well-documented influence.

Since NPI only captures the power of corporate control for the ultimate owners but not for intermediaries (such as publicly held companies), we previously were able to describe only the counter of the power distribution in the global ownership network. Now as NPF adequately measures both the distribution and flow of corporate control throughout the network, we are able to observe the landscape of the power distribution in terms of ownership influence possessed by any shareholders—the ultimate owners and intermediaries alike.

Moreover, since NPF can be interpreted as a generalized version of NPI, comparisons of NPI and NPF proves useful in several ways. First, the size of ownership influence between the different classes of shareholders can be directly compared in a coherent manner. While existing studies have emphasized the ownership influence of the financial institutions [3, 4] and state entities [28] separately, NPF allows us to compare them on a single metric. In doing so we can also assess, for example, the relative size of the influence on corporate decision-making among ultimate owners (e.g., governments), intermediaries with powerful ultimate owners (e.g., state-owned enterprises), and intermediaries with weak ultimate owners (e.g., financial institutions).

Second, although NPI and NPF are highly correlated with each other at the macro level, the disparity is also substantial at the firm level. The disparity often helps to detect excessive practices of cross-ownership and to characterize the extent to which intermediate shareholders’ ownership influence is independent of their ultimate owner’s.

Finally, just like NPI, since NPF also concerns corporate control that can be established often with a majority share, the amount of ownership share that flows through a shareholder is not necessarily linear in the amount of the influence the shareholder holds over the corporate control (measured by NPF). This non-linearity is also amplified by the network-effect. Consequently, although there is a strong correlation between PageRank (that measures the flow of equity shares) and NPF at the macro level, there is also a large disparity. Close examinations of this disparity helps to identify the type of shareholders with ownership patterns that yeild exceptionally high rate of obtaining corporate control relative to the amount of ownership share.

Despite these promises, more work is needed to make the NPI and NPF models and their asscoaited algorithms useful in practice. One crucial limit in this study is the incomplete coverage and representativeness of the available data on ownership [31]. As we mentioned above, we have addressed this problem by adopting the simplifying assumption in accordance with the seminal work by Berle and Means [32]. To make our models and algorithms more robust and viable, we will relax this assumption by using an alternative imputation strategy suggested by Leech in the future investigation of the global network of corporate ownership and control [34].

## Supporting information

S1 Appendix Formal definitions of NPI and NPF. (PDF)

S2 Appendix Ownership structure in BlackRock and Vanguard group. (PDF)

S3 Appendix How is NPF different from Betweenness Centrality? (PDF)
