# Supplementary material for: The flow of corporate control in the global ownership network
Source: PLoS One. 2023 Aug 24;18(8):e0290229. doi: 10.1371/journal.pone.0290229 (PMC10449170; doi:10.1371/journal.pone.0290229)
Supplement: S1 Appendix — (PDF) [file pone.0290229.s001.pdf]

# S1 Appendix. Formal definitions of NPI and NPF

We formally describe the model of NPF in a generalized setting. Consider an ownership network that is a graph $G = (N, X, q, v)$, where $N = \{1, 2, \ldots, n\}$ is a set of entities (i.e., companies and shareholders, individual and institutional alike), $X$ is a matrix of ownership shares $x_{ij} \in [0, 1]$ in company $j$ owned by shareholder $i$, $q$ is a vector of quotas, $q_j \in [1/2, 1]$, per company $j$'s decision rule, and $v$ is a vector of the value of companies $j$ (e.g., the sales or the number of employees).

Following the Shapley-Shubik power index, we let $N_j = \{i \in N \mid x_{ij} > 0\}$ denote a set of shareholders holding ownership in company $j$ and $S_j \subseteq N_j$ denote a coalition of shareholders (i.e., a permutation of $i \in N_j$). If the sum of voting rights amassed in a coalition exceeds the quota of company $j$, i.e., $\sum_{i \in S_j} x_{ij} > q_j$, this coalition is called a winning coalition in $j$ and denoted by $W_j$. If shareholder $i$ belongs to a winning coalition and its withdrawal breaks this coalition, i.e., if $S_j \in W_j$ and $S_j \backslash \{i\} \notin W_j$, then shareholder $i$ is a *pivot* in $S_j$ who, as Shapley and Shubik argue, controls decision-making in $j$, and hence establishes a (direct) control linkage denoted by $y_{ij} = 1$. Also following Shapley and Shubik, we assume that every coalition $S_j$ available in $N_J$ may be realized with equal probability. Then, the next definition follows:

**Definition 1** (The Shapley-Shubik power index)**.** *Shareholder i's power of controlling j is given by the probability that i is a pivot in $S_j$ such that*

$$p_{ij}(N_j, x_j, q_j) \equiv P(y_{ij} = 1 \mid W_j) = \sum_{S_j \in W_j, S_j \backslash \{i\} \notin W_j} \frac{(s_j - 1)!(n_j - s_j)!}{n_j!}, \tag{1}$$

where $s_j$ and $n_j$ are the size of $S_j$ and $N_j$, respectively.

We have generalized the Shapley-Shubik in a network setting by taking into account various ways in which shareholder $i$ may obtain indirect control linkages in $j$ through the complex network of shareholding [1]. Let $\bar{y}_{ij} \in \{0, 1\}$ denote the indirect control relation between $i$ and $j$, where $\bar{y}_{ij} = 1$ indicates that shareholder $i$ holds an indirectly control linkage in company $j$. One way in which $i$ establishes an indirect control linkage in $j$ is by transitively connecting multiple direct control linkages $y_{ij}$. More specifically, suppose that shareholder $i$ does not have a direct control linkage to $j$ (i.e., $y_{ij} = 0$) but it does to $k$ (i.e., $y_{ik} = 1$). If shareholder $k$ has a direct control linkage to $j$ (i.e., $y_{kj} = 1$), then $i$ can establish an indirect control linkage to $j$ (i.e., $\bar{y}_{kj} = 1$), by sequencing its voting power from $I$ to $k$ and then to $j$, using $k$ as an intermediary. Letting $\bar{Y}_j$ denote a set of linkages that has indirect control $j$, we make the following assumption about behavior of a winning coalition that controls $j$:

**Assumption 1** (Union formation)**.** *If shareholders k are under the control of ultimate owner i (i.e., $\bar{y}_{ik} = 1$) and have ownership in j (i.e., $x_{kj} > 0$), then these shareholders form a union $U^i_j(\bar{Y}_j) \subseteq N_j$ as a permutation of the entities k.*

This means that, as we discussed in the main text using the example of the majority coalition consisting of two

shareholders $A$ and $D$ over company $E$, there are two possible ways in which $A$ and $D$ can form a union, $\{A, D\}$ and $\{D, A\}$. That is the order in which a shareholder joins a union matters since per the Shapley-Shubik power index, the power to control a union is given to a pivotal voter that address the question of who is the last piece of the elements in a union for the union to form the winning (majority) coalition?

Moreover, for every union that controls $j$, we maintain Assumption 1 about voting coordination. Then the next corollary follows from Assumption 1.

**Corollary 1** (Integrity of Unions)**.** *Every union must maintain integrity so that any member of an union cannot defect from its union to join another in order to form a winning coalition.*

That is, any shareholder is prohibited from breaking its union to join another union in order to be a pivot in a new winning coalition. Every union must maintain integrity and any member of an union cannot defect from its union to join another in order to form a winning coalition. Since any union cannot cut across multiple winning coalitions in $j$, a family of winning coalitions must subsume the entirety of any given union within themselves $W_j(\bar{Y}_j)$.

With these assumptions about voting behavior and the type of winning coalitions being permitted, we now define an *individual* NPI of $i$ against $j$ in a network $G = (N, X, q, v)$ as the probability that ultimate owner $i$ forms an indirect control linkage to $j$:

**Definition 2** (Network Power Index)**.**

$$\bar{p}_{ij}(G) \equiv P(\bar{y}_{ij} = 1) \tag{2}$$

$$= \sum_{\bar{Y}_j} P(\bar{Y}_j) \cdot P(\bar{y}_{ij} = 1 \mid \bar{Y}_j) \tag{3}$$

$$= \sum_{\bar{Y}_j} P(\bar{Y}_j) \left( \sum_{k \in U_j^i(\bar{Y}_j)} P(y_{kj} = 1 \mid W_j(\bar{Y}_j)) \right), \tag{4}$$

where $P(y_{kj} = 1 \mid W_j(\bar{Y}_j))$ is the probability that intermediate shareholder $k$ is a pivot in $S_j$ given unions under an indirect control structure $U_j^i(\bar{Y}_j)$.

Note that we define this probability by substituting $W_j(\bar{Y}_j)$ in the Shapley-Shubik Power Index in Eq. (1). This is the key element in extending their original power index to the ownership network because ultimate owner $i$ indirectly controls target company $j$ if an intermediary $k$ under the control of $i$ becomes a pivot and, moreover, any winning coalition can have at most a single pivot in the definition of the Shapley-Shubik power index.

An important and useful characteristic of NPI is that the sum of individual NPI values among all the ultimate owner for each target company $j$ is equal to one (1). As we show in Proposition 1 below in this Appendix, this is guaranteed by the claim that individual NPI is Pareto optimal.

While individual NPI $\bar{p}_{ij}(G)$ measures the extent to which an ultimate owner has the power to influence the

managerial decision-making in a specific target company $j$, aggregated NPI sums up all the individual NPI values possessed by $i$ in the network and measures its overall power to influence all the companies. We call this quantity aggregate NPI which is given by $\bar{p}_i(G) \equiv \sum_j \bar{p}_{ij}(G)$. As for interpretation, since ultimate owner $i$'s individual NPI with respect to $j$ gives the probability that $i$ controls $j$, aggregate NPI, that is, the sum of all individual NPIs for $i$ gives the expected number of companies this ultimate owner may indirectly control in the entire network.

We can also weight individual NPI as well as aggregate NPI by the value of a target company. As a corollary, the sum of NPIs weighted by the value of a target company, $v_j$, is the expected value under its control. We call this quantity an *aggregate* NPI, denoted by $\bar{p}_i(G) = \sum_j v_j \bar{p}_{ij}(G)$.

Now that we have defined NPI, we are ready to define NPF, that is the power of intermediate shareholder in the ownership network. The intermediate shareholder's power is defined as the power of being the last piece in a union that makes the union become a winning coalition over a target company, which is led by its ultimate owner. That is, an intermediate shareholder's the power is derived from the fact that the shareholder is indispensable for its ultimate owner to establish a control path (i.e., a winning coalition) over a target company.

NPF for $k$ can be interpreted as the expected number of companies in the downstream below $k$ on its control paths. Suppose that we obtain a set of direct control paths $(N, Y)$ by repeatedly applying the procedures above. An element in the direct control matrix, $y_{kj}$, is one if $k$ is a pivot in $S_j$ with unions and zero otherwise. Then, given this network, the expected number of companies in the downstream of a control path, $p(Y, v) = (p_1(Y, v), \ldots, p_n(Y, v))'$, is defined by $p(Y, v) = Yp(Y, v) + v$, or $p(Y, v) = (I - Y)^{-1}v$.

If an ownership structure entails cross-ownership causing a loop in the ownership network, we instead use $p(Y, v) = (I - d \cdot Y)^{-1}v$ where $d \in (0, 1)$ is a damping factor to avoid the indeterminacy. Finally, since a direct control network is stochastic, we obtain an aggregated NPF value for company $k$ by taking the average:

**Definition 3** (Network Power Flow)**.**

$$p_k(G) \equiv \sum_Y P(Y) p_k(Y, v) = \sum_j v_j P(\bar{y}_{kj} = 1), \tag{5}$$

where $p_k(Y, v)$ is the $k$-th element of $(I - d \cdot Y)^{-1}v$. Note that if there is cross-ownership and hence no loop in the ownership structure in the network, the aggregate NPF value is equivalent to its corresponding aggregate NPI value for every ultimate owner (Proposition 2).

## Properties of NPF

In this section, we derive the properties of NPF in the case of no loops in $G$, i.e., $d = 1$. Let $Y$ denote a direct control matrix with unions, where $y_{kj}$ is one if player $k$ is a pivot in $j$ with unions $U_j^i(\bar{Y}_j)$ and zero otherwise. Similarly, let $\bar{Y}$ denote an indirect control matrix, where $\bar{y}_{ij}$ is one if $i$ establishes an indirect control of $j$ and zero otherwise.

**Lemma 1.** *$Y^n$ is a matrix such that its $ij$-th element is one if player $i$ can reach $j$ in $n$ steps and zero otherwise.*

*Proof.* Since $Y$ is an adjacency matrix, its $n$-th power, $Y^n$, indicates the number of paths between $i$ and $j$ with the length of $n$. By the definition of a pivot, there exists only one pivot for each company $j$; if $y_{kj} = 1$, then $y_{k'j} = 0, \forall k' \neq k$. Let the $ij$-th element of $Y^n$ be denoted by $y_{ij}^{(n)}$. We obtain $y_{ij}^{(n)} = \sum_{k' \in N} y_{ik'}^{(n-1)} y_{k'j} = y_{ik}^{(n-1)}$ for $k$ such that $y_{kj} = 1$. Since $y_{ij}^{(1)} \in \{0, 1\}$ holds, $y_{ij}^{(1)} \in \{0, 1\}, \forall n$ also holds by induction, implying that there exists only one path between $i$ and $j$ in $n$ links if it exists. □

This lemma implies that we can obtain the indirect control matrix $\bar{Y}$ as $(I - Y)^{-1}$.

**Lemma 2.** *An indirect control $\bar{Y}$ is equivalent to $(I - Y)^{-1}$.*

*Proof.* Following the Neumann series, we obtain $(I - Y)^{-1} = \sum_{n=0}^{\infty} Y^n = I + Y + Y^2 + \cdots$. Because we assume that there is no loop, there exists $n$ such that $(I - Y)^{-1} = \sum_{n=0}^{n} Y^n$. Following the lemma 1, $(I - Y)^{-1}$ is a reachability matrix whose element indicates whether any two players are connected or not in $(N, Y)$. This is an indirect control matrix by definition. □

The Pareto optimality of an individual NPI and the equivalence of NPI and NPF follow this lemma.

**Proposition 1** (Pareto optimality)**.** *An individual NPI is Pareto optimal; $\sum_i \bar{p}_{ij}(G) v_j = v_j$.*

*Proof.* Because the event $\bar{y}_{ij} \in \{0, 1\}$ is exclusive, $\sum_i P(\bar{y}_{ij} = 1) = 1$ holds, hence, $\sum_i \bar{p}_{ij}(G) v_j = \sum_i P(\bar{y}_{ij} = 1) v_j = v_j$. □

**Proposition 2** (Equivalence of NPI and NPF)**.** *For ultimate owner $i$, $p_i(G) = \bar{p}_i(G)$.*

*Proof.* From the lemma 2, we obtain $p(Y, v) = \bar{Y} v$, implying that $p_i(Y, v)$ is equal to $\sum_j \bar{y}_{ij} v_j$. By taking an expectation, we obtain the NPF for $i$, i.e., $p_i(G) \equiv E[p_i(Y, v)] = \sum_j E[\bar{y}_{ij}] v_j = \sum_j P(\bar{y}_{ij} = 1) v_j = \bar{p}_i(G)$, hence, the aggregate NPI. □

The proposition below ensures that the algorithm in the main text consistently estimates NPFs.

**Proposition 3** (Consistency)**.** *The estimator of NPF, $\hat{p}(G)$, converges in probability to the true value, $p(G)$, as the number iteration $T$ increases, i.e., $\hat{p}(G) \to_p p(G)$.*

*Proof.* The $ij$-the element of $\hat{p}$ is $\sum_{t=1}^{T} p_{ij}^{(t)} v_j / T$, which is equal to $\sum_{t=1}^{T} \bar{y}_{ij}^{(t)} v_j / T$ since $\bar{Y} = (i - Y)$ following the lemma 2. And, as shown in the proof of proposition 2, we have $p_i(G) = \sum_j P(\bar{y}_{ij} = 1) v_j$. These mean if $\sum_{t=1}^{T} \bar{y}_{ij}^{(t)} / T$ converges to $P(\bar{y}_{ij} = 1)$, then the argument is true. We will show $\sum_{t=1}^{T} \bar{y}_{ij}^{(t)} / T \to_p \sum_{\bar{Y}_j} P(\bar{Y}_j) \cdot P(\bar{y}_{ij} = 1 \mid \bar{Y}_j) = P(\bar{y}_{ij} = 1)$.

For an indirect control structure $\bar{Y}_j$, let $\mathcal{T}(\bar{Y}_j)$ be a set of $t$ such that $\bar{Y}_j^{(t)} = \bar{Y}_j$ and $T(\bar{Y}_j)$ be the size of $\mathcal{T}(\bar{Y}_j) \subseteq T$. Then, because the proportion of $\bar{y}_{ij} = 1$ given $\bar{Y}_j$ is $\sum_{t \in \mathcal{T}(\bar{Y}_j)} \bar{y}_{ij}^{(t)} / T(\bar{Y}_j)$ and that of $\bar{Y}_j$ is $T(\bar{Y}_j)/T$, the

frequency of $\bar{y}_{ij} = 1$ can be decomposed as follows

$$\frac{\sum_{t=1}^{T} \bar{y}_{ij}^{(t)}}{T} = \sum_{\bar{Y}_j} \underbrace{\frac{\sum_{t\in\mathcal{T}(\bar{Y}_j)} \bar{y}_{ij}^{(t)}}{T(\bar{Y}_j)}}_{(1)} \cdot \underbrace{\frac{T(\bar{Y}_j)}{T}}_{(2)}. \tag{6}$$

Therefore, if the first part and the second part converge to $P(\bar{y}_{ij} = 1 \mid \bar{Y}_j)$ and $P(\bar{Y}_j)$, then we obtain the proposition.

**Part (1)** We further decompose the first part of Eq. 6 as follows;

$$\frac{\sum_{t\in\mathcal{T}(\bar{Y}_j)} \bar{y}_{ij}^{(t)}}{T(\bar{Y}_j)} = \sum_{k\in U_j^i(\bar{Y}_j)} \frac{\sum_{t\in\mathcal{T}(\bar{Y}_j)} y_{kj}^{(t)}}{T(\bar{Y}_j)}. \tag{7}$$

Given $\bar{Y}_j$, the event of $\{y_{kj}^{(t)} \in \{0,1\}\}$ is a series of $T(\bar{Y}_j)$ times Bernoulli trial with the probability of $P(y_{kj} = 1 \mid W_j(\bar{Y}_j))$. Follwing [2], Hoeffding's inequality implies

$$P\left(\left|\frac{\sum_{t\in\mathcal{T}(\bar{Y}_j)} y_{kj}^{(t)}}{T(\bar{Y}_j)} - P(y_{kj} = 1 \mid W_j(\bar{Y}_j))\right| \geq \varepsilon\right) \leq 2e^{-2T(\bar{Y}_j)\varepsilon^2}, \tag{8}$$

which ensures

$$\frac{\sum_{t\in\mathcal{T}(\bar{Y}_j)} y_{kj}^{(t)}}{T(\bar{Y}_j)} \to_p P(y_{kj} = 1 \mid W_j(\bar{Y}_j)) \text{ as } T(\bar{Y}_j) \to \infty. \tag{9}$$

Hence, we show

$$\sum_{k\in U_j^i(\bar{Y}_j)} \frac{\sum_{t\in\mathcal{T}(\bar{Y}_j)} \bar{y}_{ij}^{(t)}}{T(\bar{Y}_j)} \to_p \sum_{k\in U_j^i(\bar{Y}_j)} P(y_{kj} = 1 \mid W_j(\bar{Y}_j)) = P(\bar{y}_{ij} = 1 \mid \bar{Y}_j). \tag{10}$$

**Part (2)** Let $N^l$ be a set of players $j$ such that the distance of the longest path from any ultimate owner to $j$ is $l$. That is, $N^0$ is the set of ultimate owners and $N^1$ is the set of copmanies whose shares are owned by only ultimate owners. For $k \in N^1$, indirect control linkage $\bar{y}_{ik}$ is equal to direct control linkage $y_{ik}$.

Then, consider company $j \in N^2$, which are likned with ultimate owners and companies $k \in N^1$. Indirect control structure above $j$, $\bar{Y}_j$, is control linkages between $i$ and $k$. Therefore, the probability of a specific control sturucture is $P(\bar{Y}_j) = \prod_k P(\bar{y}_{ik} = 1)$. On the other hand, the empirical distribution of $\bar{Y}_j$ is

$$\frac{T(\bar{Y}_j)}{T} = \sum_t \frac{\prod_k \bar{y}_{ik}^{(t)}}{T}. \tag{11}$$

This stochastically converges to $\prod_k P(\bar{y}_{ik} = 1)$ because ultimate owner $i$ being pivot in $k$ is independent for $k \in N^l$ and the probability of being a pivot is consistently estimated as discussed in part (1).

For $j' \in N^3$, an indirect control structure emerges with the probability of

$$P(\bar{Y}_{j'}) = \prod_j P(\bar{y}_{ij} = 1) = \prod_j \sum_{\bar{Y}_j} P(\bar{y}_{ij} = 1 \mid \bar{Y}_j) P(\bar{Y}_j). \tag{12}$$

The empirical distribution $T(\bar{Y}_{j'})/T$ converges in probability to this quantity because $P(\bar{y}_{ij} = 1 \mid \bar{Y}_j)$ and $P(\bar{Y}_j)$ are also consistently estimated. The fact the probability of indirect control structure $\bar{Y}_{j'}$ can be consistently estimated for $j' \in N^3$ ensures the probability of $\bar{Y}_j$ for $j \in N^4$ is also possible to consistently estimate and so forth. Hence, by induction, we can show for $j \in N^l, \forall l$

$$\frac{T(\bar{Y}_j)}{T} \to_p P(\bar{Y}_j) \text{ as } T \to \infty. \tag{13}$$

Finally, since $T$ increases $T(\bar{Y}_j)$, we have

$$\frac{\sum_{t=1}^{T} \bar{y}_{ij}^{(t)}}{T} \to_p \sum_{\bar{Y}_j} P(\bar{y}_{ij} = 1 \mid \bar{Y}_j) P(\bar{Y}_j) = P(\bar{y}_{ij} = 1), \tag{14}$$

or $\hat{p}(G) \to_p p(G)$. □

# References


[1] Mizuno T, Doi S, Kurizaki S. The Power of Corporate Control in the Global Ownership Network. PLOS One. 2020;15(8):e0237862.

[2] Bachrach Y, Markakis E, Resnick E, Procaccia AD, Rosenschein JS, Saberi A. Approximating power indices: theoretical and empirical analysis. Autonomous Agents and Multi-Agent Systems. 2010;20(2):105–122.
