# Supplementary material for: The flow of corporate control in the global ownership network
Source: PLoS One. 2023 Aug 24;18(8):e0290229. doi: 10.1371/journal.pone.0290229 (PMC10449170; doi:10.1371/journal.pone.0290229)
Supplement: S2 Appendix — (PDF) [file pone.0290229.s002.pdf]

## S2 Appendix. Ownership structure in BlackRock and Vanguard group

**Independence of Vanguard Group:** The Vanguard Group Inc. is the "parent company" of all the Vanguard funds, which is privately held fully by its own funds alone; it is not owned by any non-Vanguard entity. This unique ownership structure allows Vanguard Group Inc. to maintain the independence from any entities' influence in the global ownership market. Table 1 lists all the ultimate owners of Vanguard Group Inc. and their individual NPI values over Vanguard Group Inc. Vanguard Group Inc. is ultimately owned exclusively by its own funds, where individual NPI values vis-a-vis the Vanguard Group Inc. held by these five ultimate owners sum exactly up to one (1).

**Independence of BlackRock:** Unlike Vanguard, BlackRock is a publicly-listed company and widely held, Table 2 lists its fifteen largest ultimate owners and their individual (non-weighted) NPI values over BlackRock. The top 20 ultimate owners collectively occupy roughly 75% of individual NPI over BlackRock, leaving only 0.25 for all other shareholders with the ultimate ownership of BlackRock. There are several interesting characteristics emerging from this table. First none of these top 20 ultimate owners is a single dominant power to control BlackRock so that it is safe to say that BlackRock is fairly independent of the influence from other entities in exercising its power to control the economy. Second, these top 20 companies overlap the most influential shareholders overall in the global ownership network shown in Table 1 in the main text. Third, none of the political entities among the most influential ultimate owners appear on this list unless those political entities run sovereign wealth funds. This include for example the government of PRC and Russian Federation.

Table 1: Who Owns Vanguard in December 2020?

| | Ultimate Owners of Vanguard Group Inc. | NPI(*1) |
|---|---|---|
| 1 | Vanguard Index Funds | 0.213 |
| 2 | Vanguard Star Funds | 0.211 |
| 3 | Vanguard Bond Index Funds | 0.197 |
| 4 | Vanguard Fixed Income Securities Funds | 0.192 |
| 5 | Vanguard Institutional Index Funds | 0.188 |

Table 2: Who Owns BlackRock in December 2020?

| | Ultimate Owners of BlackRock | NPI(*1) |
|---|---|---|
| 1 | Government of Kuwait | 0.136 |
| 2 | Capital Group Companies Inc. | 0.098 |
| 3 | Johnson Family | 0.095 |
| 4 | Capital World Investors | 0.053 |
| 5 | Wellington Management Group LLP | 0.046 |
| 6 | Government of Singapore | 0.045 |
| 7 | Vanguard Bond Index Funds | 0.036 |
| 8 | Vanguard Star Funds | 0.035 |
| 9 | Vanguard Index Funds | 0.033 |
| 10 | Vanguard Fixed Income Securities Funds | 0.029 |
| 11 | Vanguard Institutional Index Funds | 0.028 |
| 12 | Government of Norway | 0.027 |
| 13 | Geode Holdings Trust | 0.024 |
| 14 | Royal Bank of Canada | 0.012 |
| 15 | Mr. Stephen McCarthy Goddard | 0.010 |
| 16 | Dodge & COX | 0.009 |
| 17 | TIAA Board of Oversees | 0.009 |
| 18 | Warren Buffett | 0.008 |
| 19 | State of California | 0.007 |
| 20 | Fayez Shalaby Sarofim | 0.007 |
| – | All other ultimate owners | 0.254 |

*Note*:(*1) Individual, non-weighted NPI over Vanguard Group and BlackRock, respectively.
