# Supplementary material for: The flow of corporate control in the global ownership network
Source: PLoS One. 2023 Aug 24;18(8):e0290229. doi: 10.1371/journal.pone.0290229 (PMC10449170; doi:10.1371/journal.pone.0290229)
Supplement: S3 Appendix — (PDF) [file pone.0290229.s003.pdf]

S3 Appendix. How is NPF different from Betweenness Centrality?

We now turn to the comparison of NPF to "betweenness centrality (BC)" that is another widely used measure for the importance of intermediaries in the linkage path(s) in a network. If a shareholder has a large influence over companies in the network of ownership in companies, then the shareholder is expected to have a large value of NPF and/or BC.

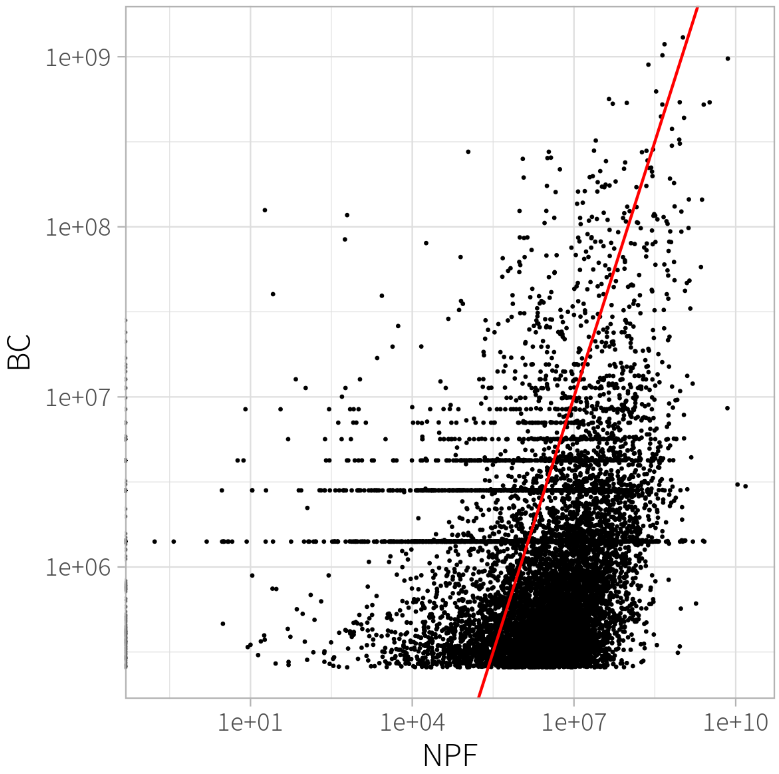

Figure 1: Comparing NPF to Betweenness Centrality

Figure 1 displays the distribution of the shareholders with BC value on the x-axis and NPF on the y-axis. Note that the scales are different on these axes, and that NPF values are weighted by the companies sales but BC values are not weighted. The red line indicates the 45-degree line. The majority of shareholders are located below the 45-degree line, meaning that the shareholders with large influence over decision-making in companies tend to have smaller BC values. This is because a node in a network tends to have a large BC value if the node has a large number of other nodes on the linkage path(s) both upstream and downstream. Thus, if a shareholder has a high NPF value, that is because the shareholder is either ultimate owner or is situated upstream in the hierarchical network of ownership, so that even if this shareholder has controlling ownership in a large number of companies downstream, its BC value is expected to be small because only a small number of shareholders are expected to be found in its upstream.

Among the categories of the shareholders we introduced earlier in terms of the degree of autonomy in exercising

Table 1: Shareholders with Top 10 Betweenness Centrality in December 2020

	Shareholders	BC	NPI**	NPF***
1	HSBC Holdings	1,298,571,445	0.00	1155.92
2	Ping An Insurance	1,184,431,828	0	526.96
3	CITIC Securities	1,059,658,761	0	-950.53
4	Schroders	1,019,448,434	0.00	481.56
5	BlackRock	975,275,460	0.02	7870.31
6	Assicurazioni Generali	898,236,037	0	264.59
7	Guotai Asset Management	846,146,971	0	-6.473
8	Orix Corp.	624,917,700	0.00	367.76
9	Mediobanca	563,841,296	0	49.18
10	Invesco	540,264,516	0	1008.66

Note:

BC: Betweenness Centrality, non-weighted

NPI: Aggregated NPI weighted by operating revenue in billion U.S. dollars

NPF: Aggregated NPF weighted by operating revenue in billion U.S. dollars

the power of corporate control, the only one category, that is the widely-held companies, such as BlackRock and State Street, may have high values both in NPF and BC simultaneously. As you can see in Table! 1 that lists top 10 shareholders with the highest BC values, there is no strong correlation between the values of BC and NPF. The only discernible pattern in this table is that the shareholders with high values on BC have very small power of corporate control. Their NPI values are either zero or negligibly small, which means that these shareholders are not located in upstream of the ownership network. Hence, BC can meaningfully measure the importance of a company in a certain type of networks such as a supply chain network of the battery production but not in a ownership network for automobile production company.
